# Supplementary material for: Genomics of soil depth niche partitioning in the Thaumarchaeota family Gagatemarchaeaceae
Source: Nat Commun. 2023 Nov 11;14:7305. doi: 10.1038/s41467-023-43196-0 (PMC10640624; doi:10.1038/s41467-023-43196-0)
Supplement: Supplementary file 1 — Supplementary Information [file 41467_2023_43196_MOESM1_ESM.pdf]

Supplementary Information: Genomics of soil depth niche partitioning in the Thaumarchaeota family Gagatemarchaeaceae

Paul O. Sheridan[1,2], Yiyu Meng[1], Tom A. Williams[3], Cécile Gubry-Rangin[1,#]

Supplementary Note 1: Classifications

Candidatus "*Gagatemarchaeum stordalenia*" (sp. nov., gen. nov). "Gagatem" refers to its prevalence in peat environments. The name "stordalenia" originates from the geographical origin (Stordalen Mire, Sweden) of the reference (type) genome, bog-1369. It encodes genes for aerobic respiration and likely uses organic substrates, such as carbohydrates, peptides and fatty acids for organoheterotrophic growth. It is currently not cultured and known only from environmental sequencing. Genomes of this genera possess a high GC-content (around 60%) and genome size of its members range from 2.2-3.4 Mb.

Candidatus "*Subgagatemarchaeum marcellia*" (sp. nov., gen. nov). "Subgagatem" refers to its prevalence in subsoils and peat environments. The name "'marcellia" originates from the geographical origin (Marcell Experimental Forest, MN, USA) of the reference (type) genome, Fn1. It likely uses organic substrates, such as carbohydrates, peptides and fatty acids for organoheterotrophic growth. While the type genome, Fn1, encodes the genes for microaerophilic respiration cytochrome bd ubiquinol oxidase, the majority of studied genomes from this genera encode the aerobic respiration heme-copper oxygen reductases. It is currently not cultured and only known from environmental sequencing. Genomes of this genera possess a high GC-content (around 58%) and genome size of its members range form 1.2-2.5 Mb.

Description of Gagatemarchaeaceae (fam. nov). This family was previously referred as the Group I.1c Thaumarchaeota. Description is the same as for the genus *Gagatemarchaeum*. Suff. -aceae, ending to denote family. Type genus *Gagatemarchaeum* gen. nov.

Supplementary Figures

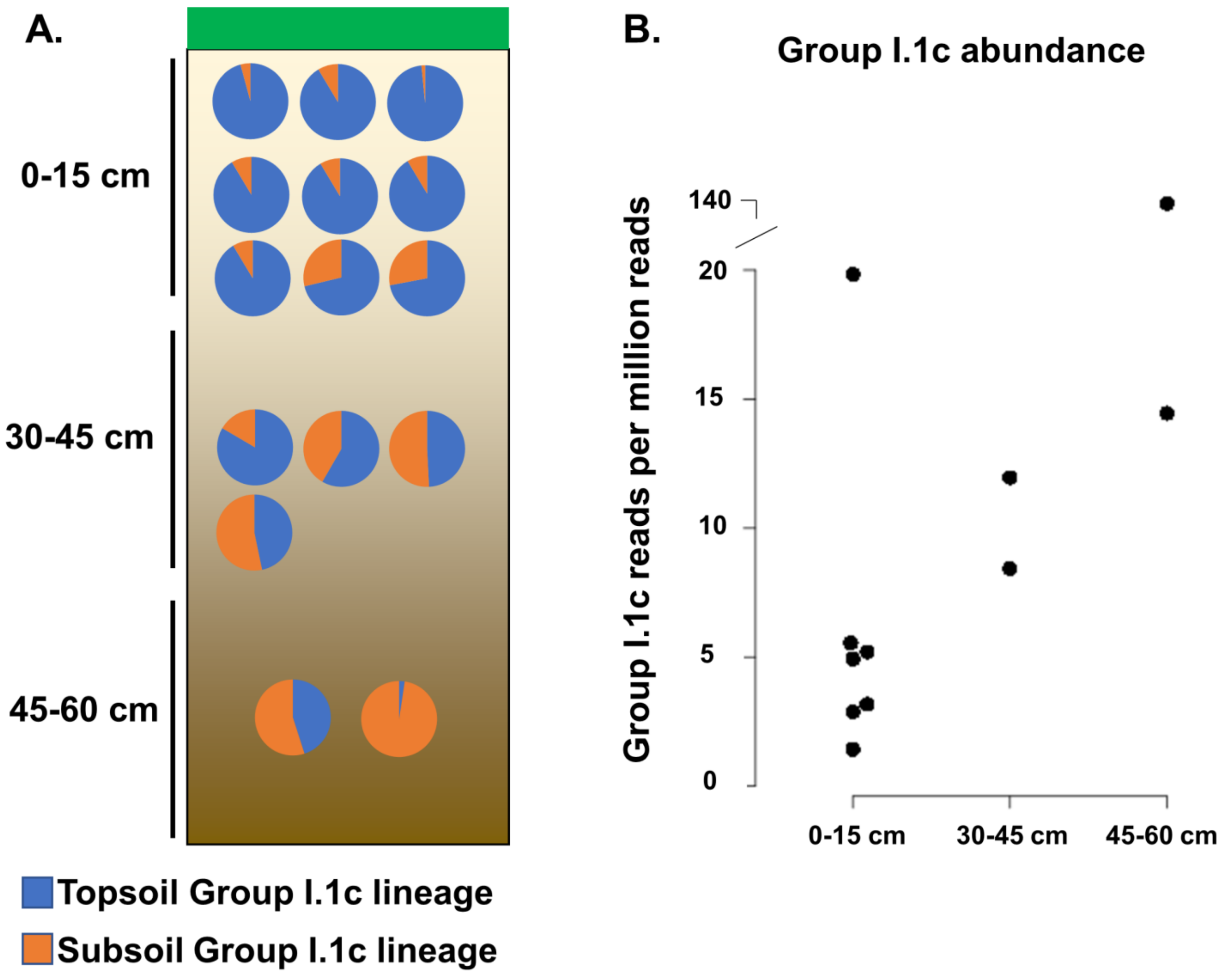

Supplementary Fig. 1. Depth distribution of soil Group I.1c lineages. (A) The Group I.1c community composition changes from Topsoil lineage dominated to Subsoil lineage dominated with increasing depth, and (B) the overall abundance of Group I.1c increases with depth. Metagenomic reads were recruited to genomes of the Topsoil and Subsoil lineages. Source data are provided as a Source Data file..

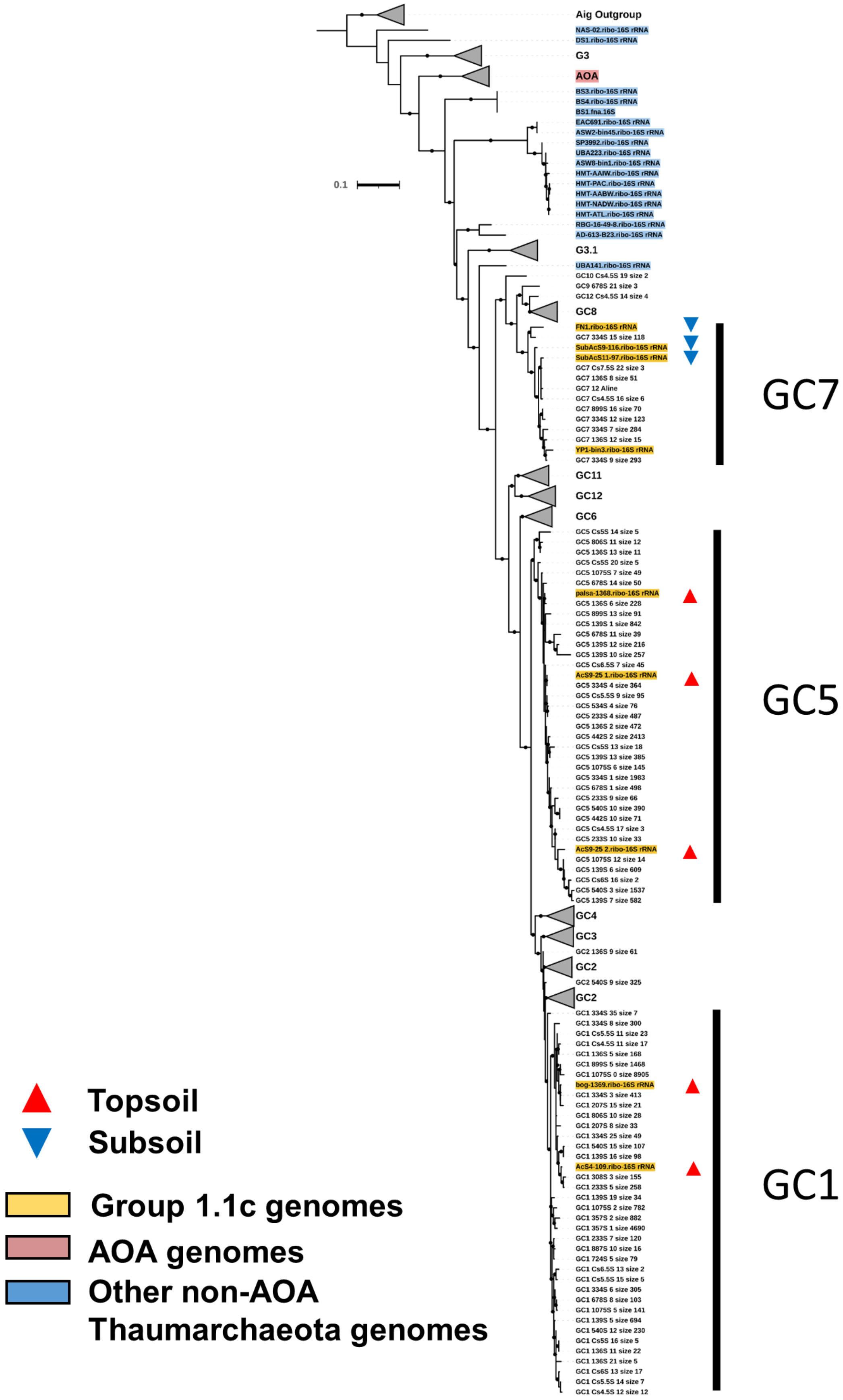

Supplementary Fig. 2. 16S rRNA gene tree of Group I.1c and related sequences. This maximum-likelihood tree was created using 16S rRNA genes extracted from the studied genomes and combined with the 16S rRNA database of soil Thaumarchaeota presented in Vico Oton et al 2016[1] (587 column alignment). Dots indicate branches with >70% of 1,000 UFBoot replicates.

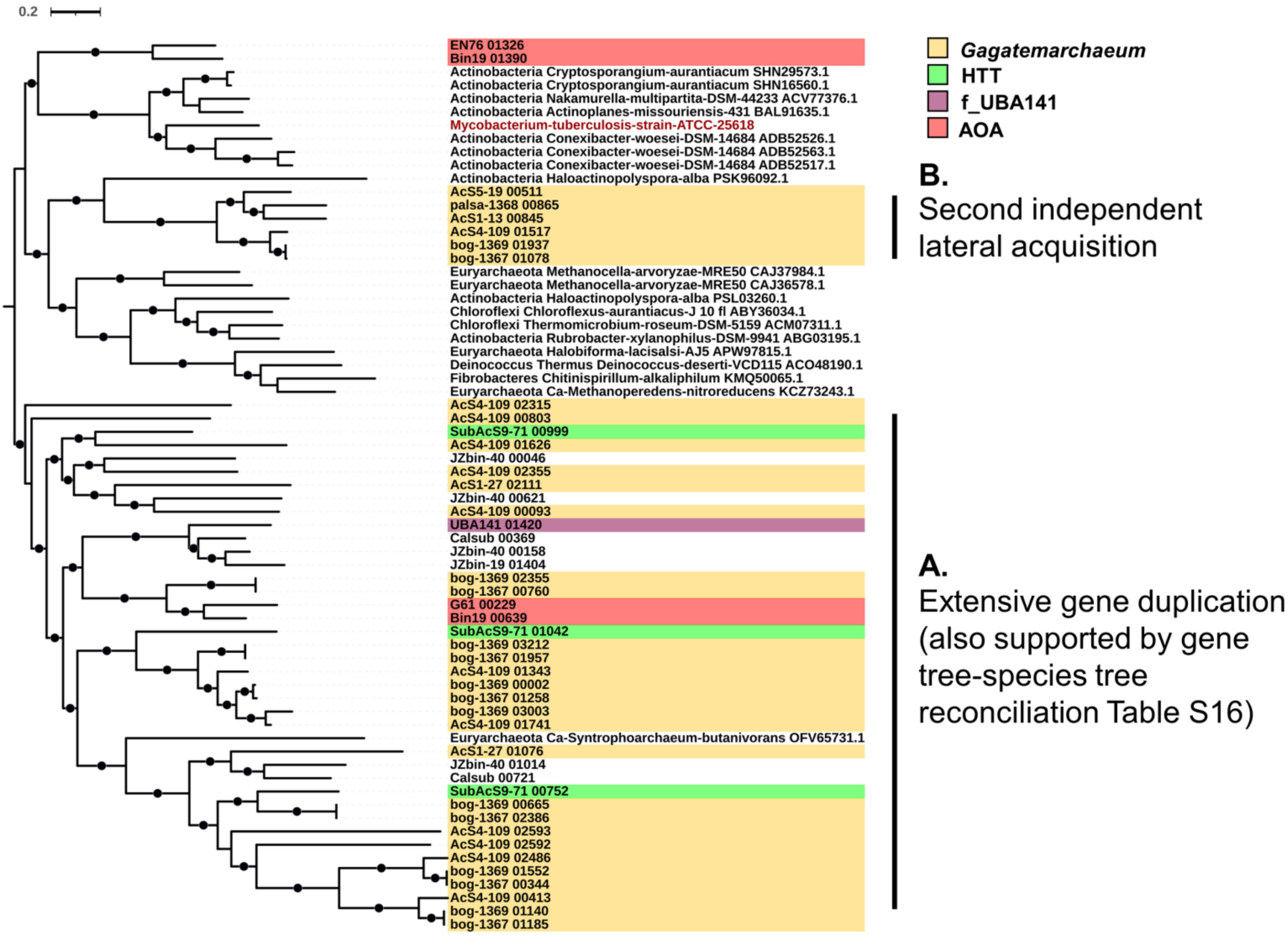

Supplementary Fig. 3. Phylogeny of F420-dependent glucose-6-phosphate dehydrogenase. This gene family was present in multiple copies in the topsoil Group I.1c genomes, owing to (A) extensive gene duplication throughout the evolution of *Gagatemarchaeum* and (B) a second independent lateral acquisition. The leaf in red is the experimentally validated gene. Dots indicate branches with >70% of 1,000 UFBoot replicates. The ML tree was created using LG+R4 and rooted with minimal ancestor deviation (MAD).

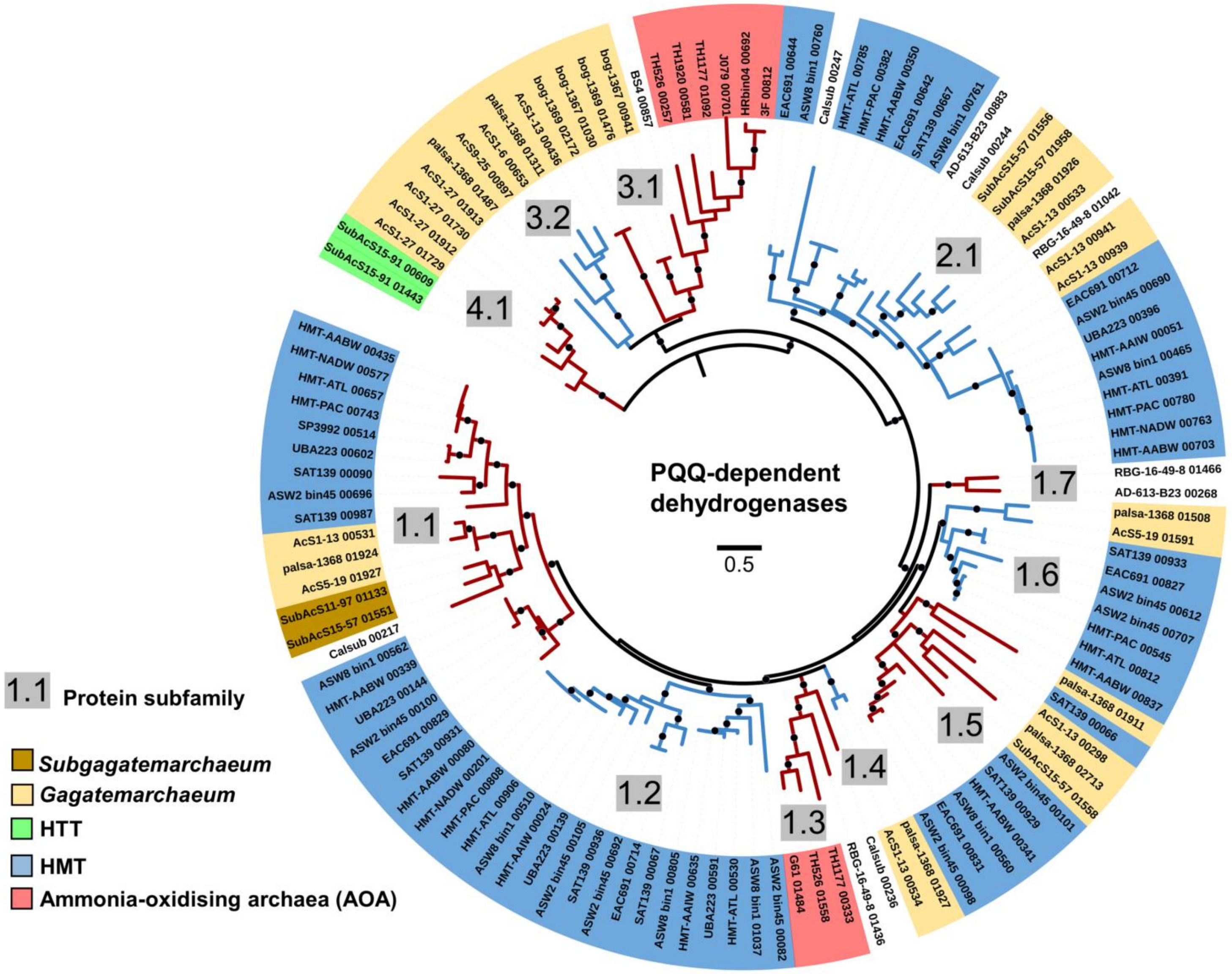

Supplementary Fig. 4. Phylogeny of Thaumarchaeota PQQ-dependent dehydrogenases. Dots indicate branches with >70% of 1,000 UFBoot replicates**.** The alternating blue and red branches indicate different subfamilies as determined by average pairwise distance between leaves. Tree was rooted using minimal ancestor deviation (MAD).

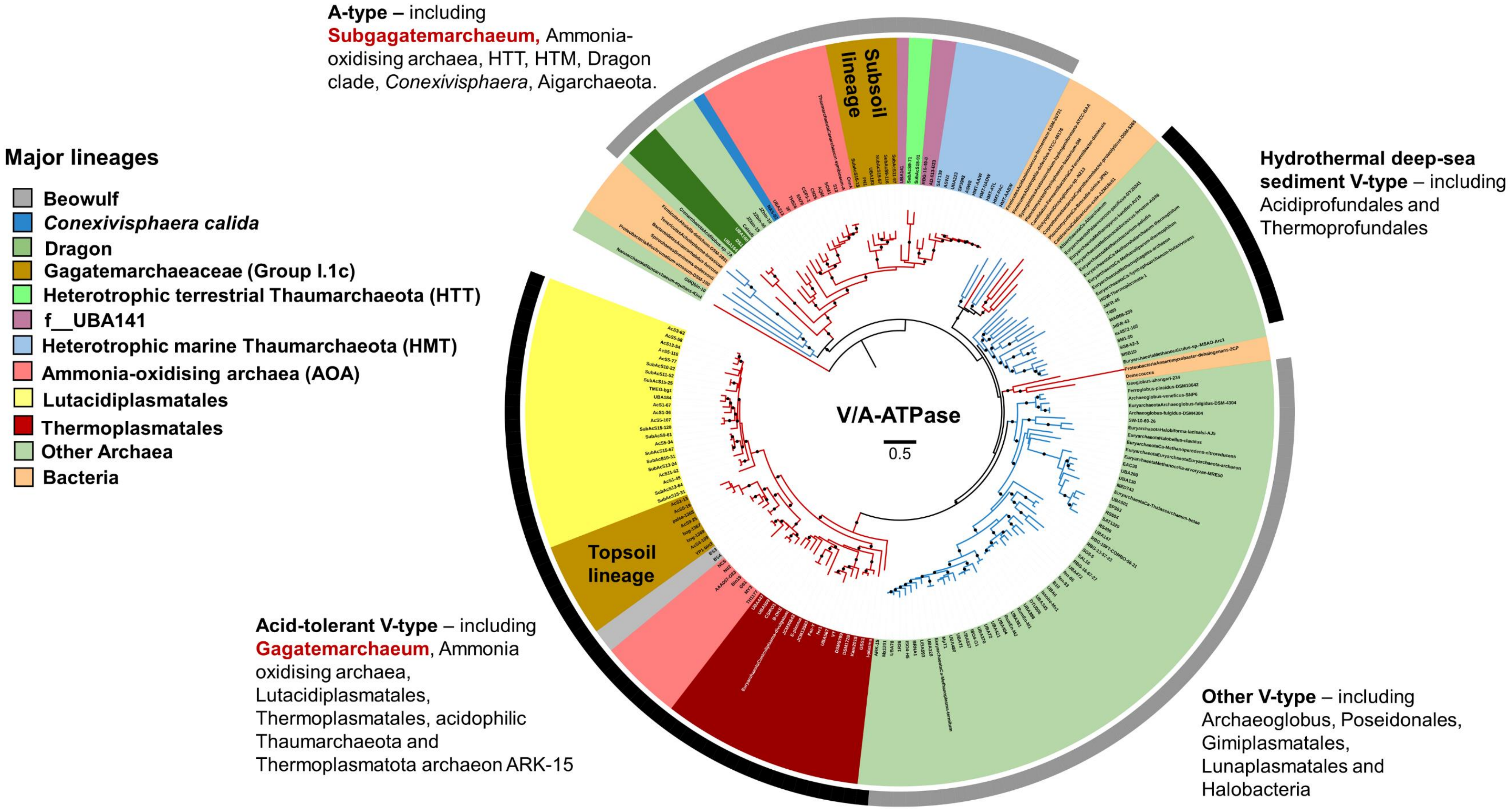

Supplementary Fig. 5. Phylogeny of the V/A-ATPase. Sequences from *Gagatemarchaeum* clustered with acid tolerant archaea, whereas those of *Subgagatemarchaeum* clustered with HMT, HTT and non-acidophilic AOA. The three largest subunits of V/A-ATPase (*atpA*, *atpB* and *atpI*) were individually aligned and then concatenated into a single partitioned supermatrix. A supermatrix tree was then estimated using the best fitting model for each partition and rooted using minimal ancestor deviation (MAD). Dots indicate branches with >70% of 1,000 UFBoot replicates. The alternating blue and red branches indicate different subfamilies of V/A-ATPase as determined by average pairwise distance between leaves.

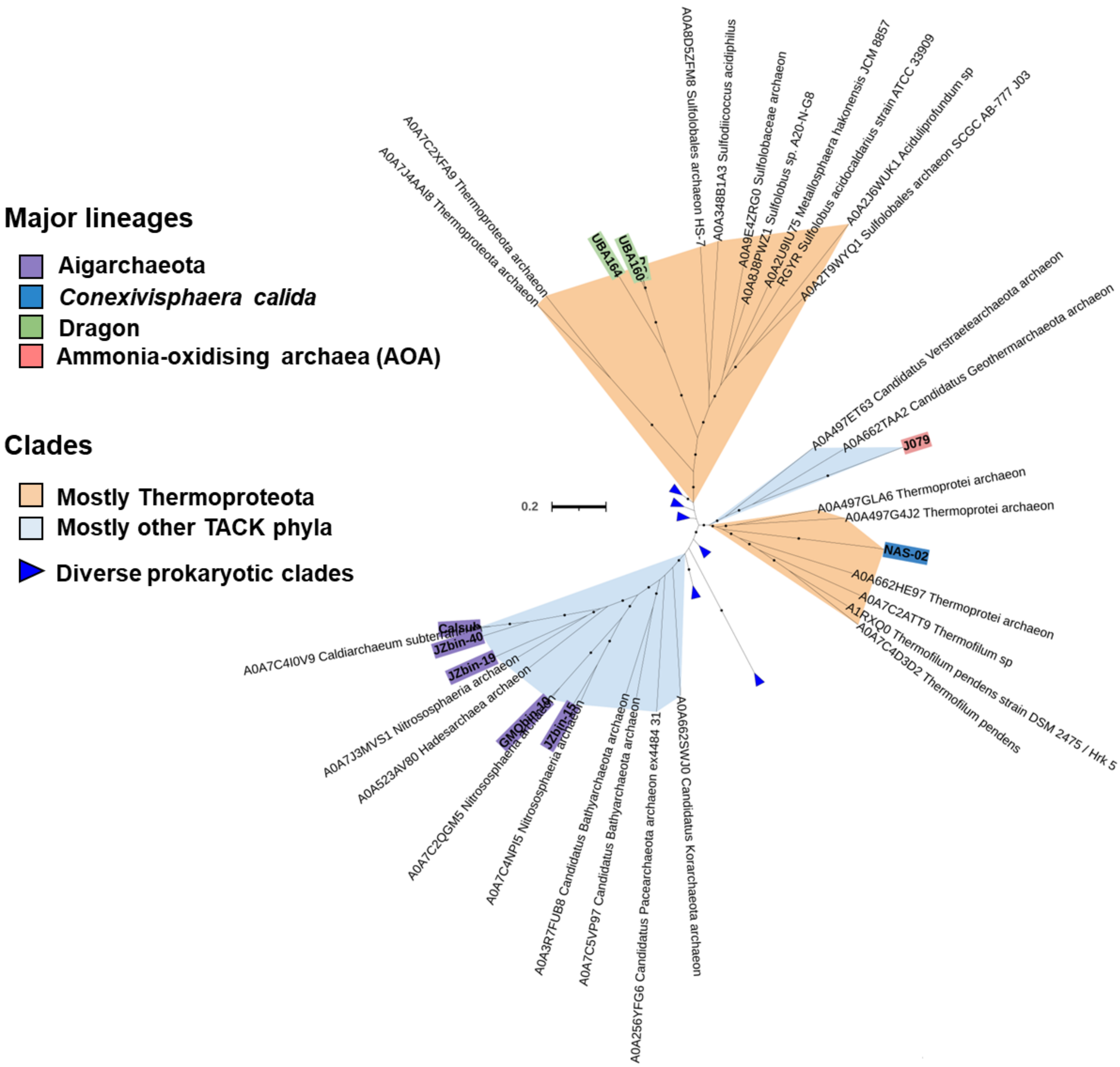

Supplementary Fig. 6. Phylogeny of Thaumarchaeota reverse gyrase, rgy. Dots indicate branches with >70% of 1,000 UFBoot replicates**.** The ML tree was predicted using the LG+F+R10 model.

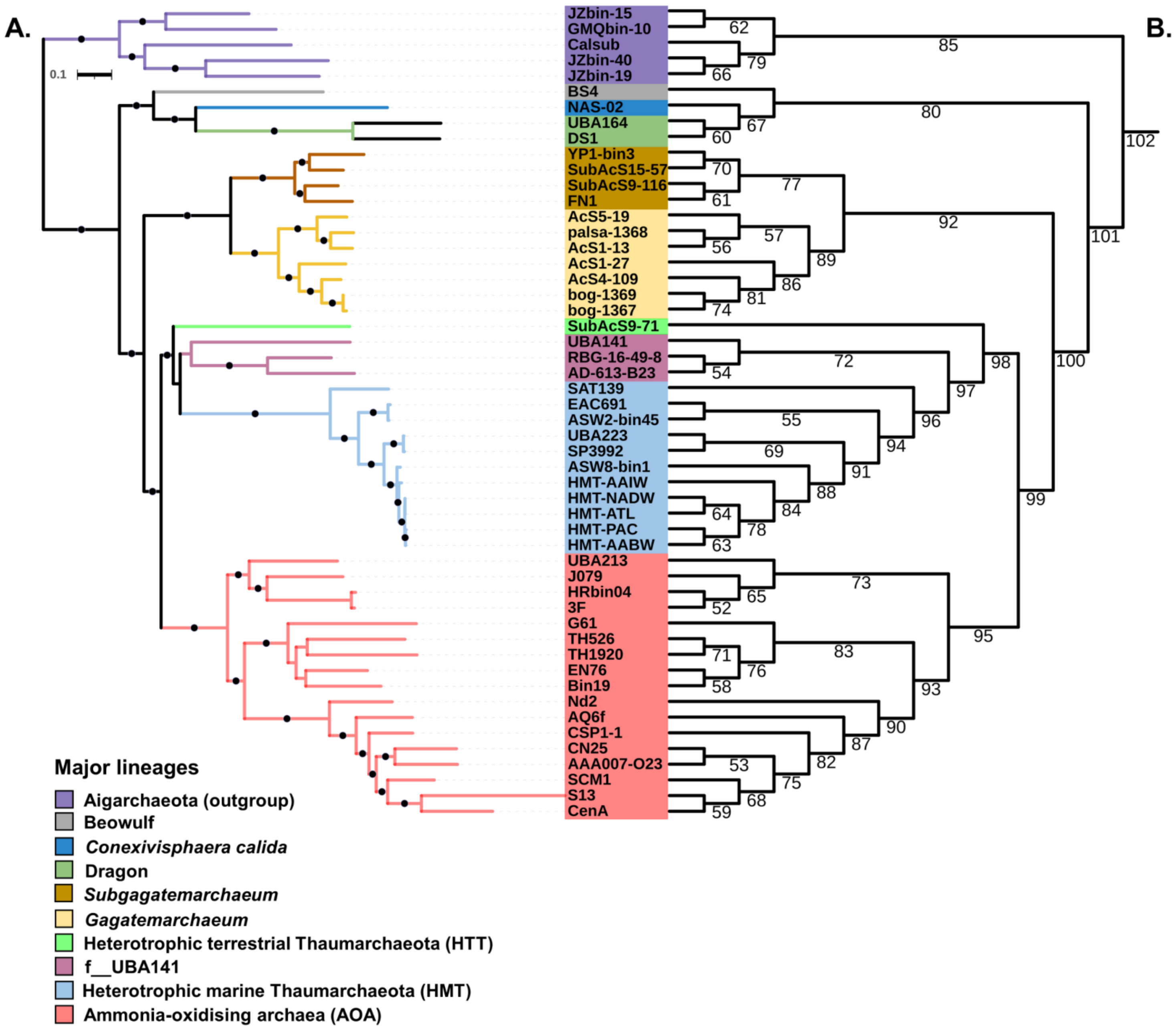

Supplementary Fig. 7. Phylogeny of higher-quality genomes (A) and corresponding branch numbers (B). The ML tree was predicted with the LG+C60+G+F model. Dots indicate branches with >95% of 2,000 UFBoot and 1,000 SH-aLRT replicates**.**

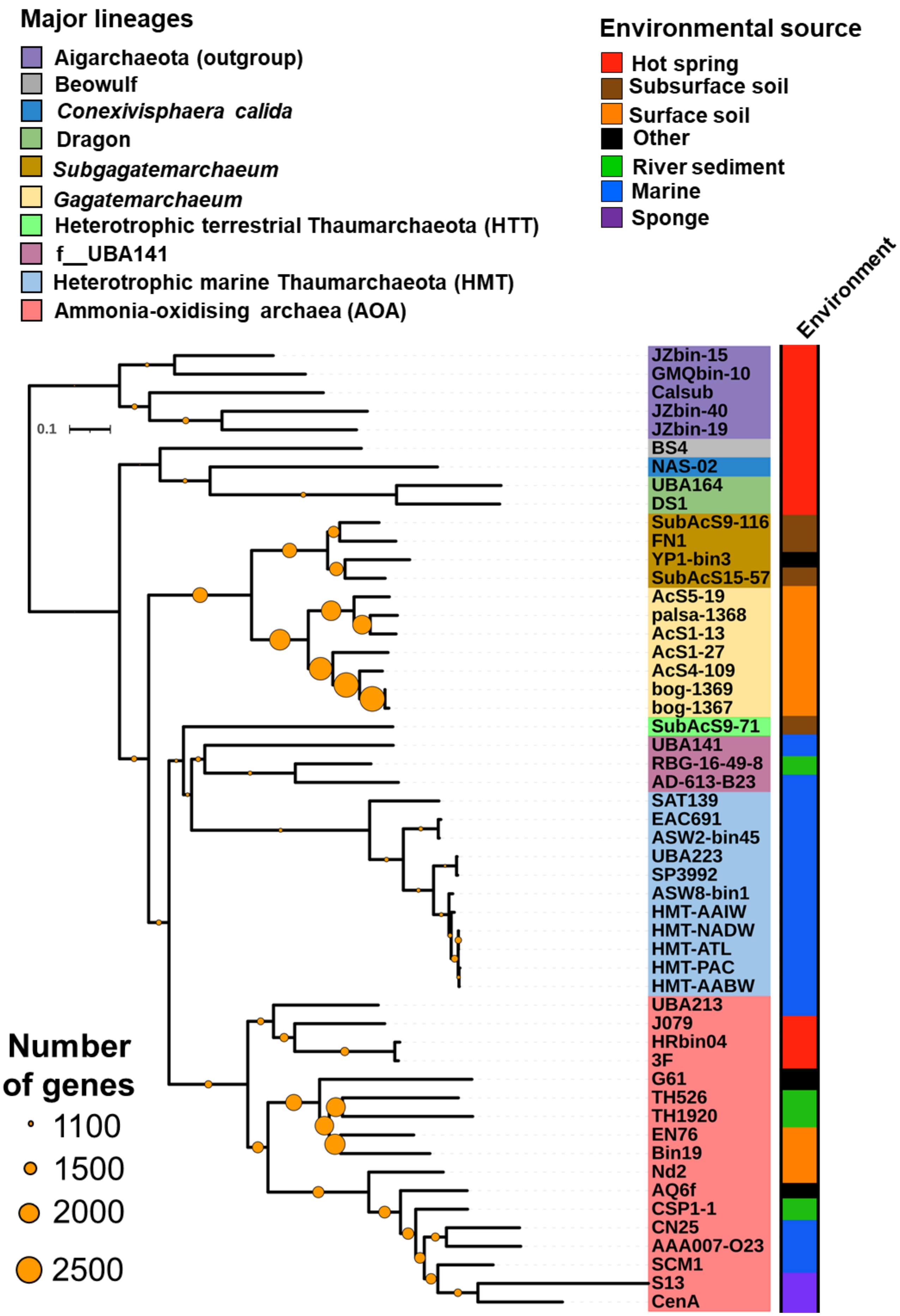

Supplementary Fig. 8. Genome expansion transitioning into terrestrial environments. Copy number of ancestor gene content reconstructions estimated using gene tree-species tree reconciliation are indicated.

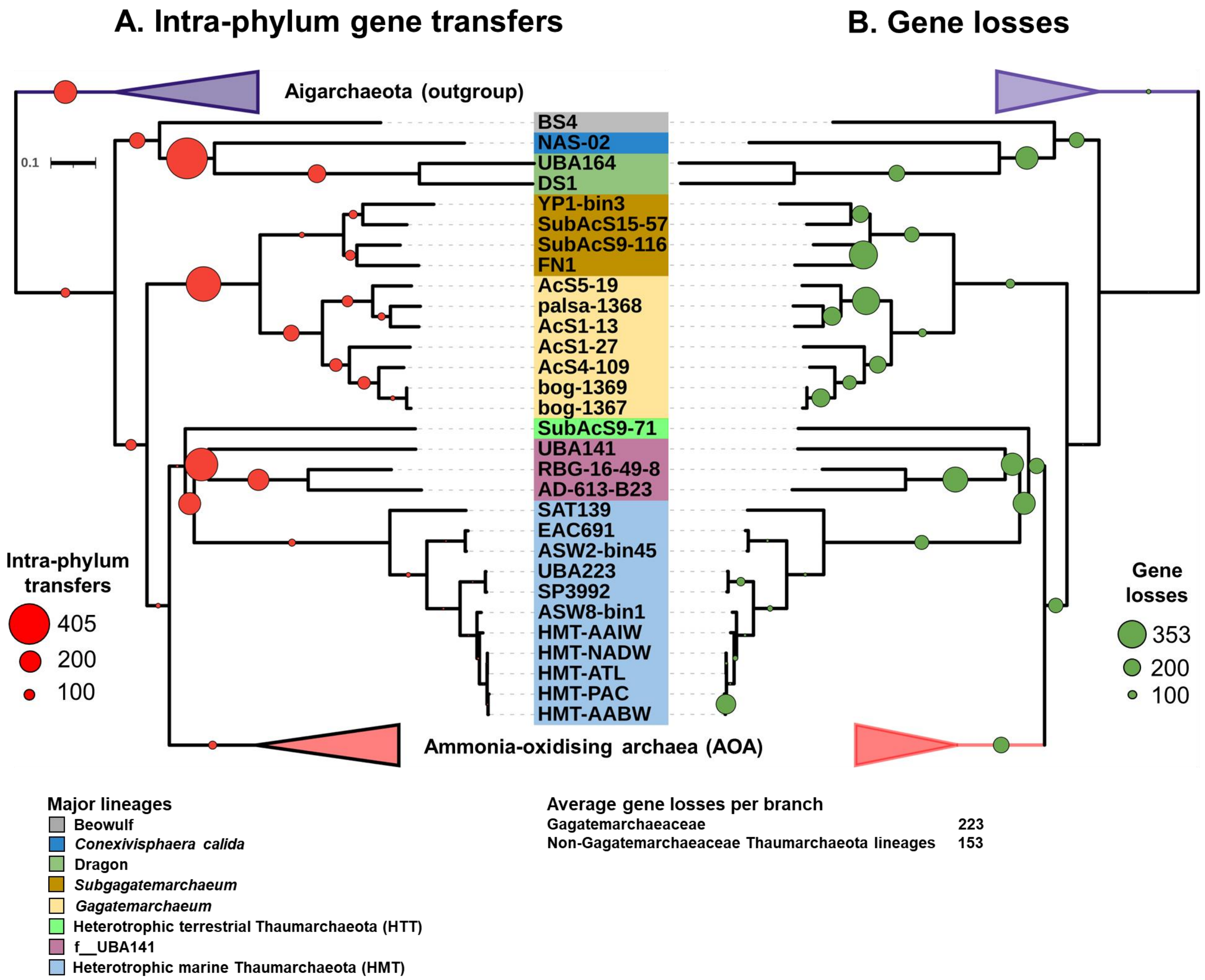

Supplementary Fig. 9. Acquisition of genes by intra-phyla transfer and gene losses along non-AOA lineages. The quantitative and qualitative predictions of intra-phyla gene transfers (A) and gene loss (B) were estimated across the Thaumarchaeota history using a gene tree-species tree reconciliation approach. Scale numbers indicate the range of the predicted number of events for a given mechanism and circle sizes are proportional to the number of events.

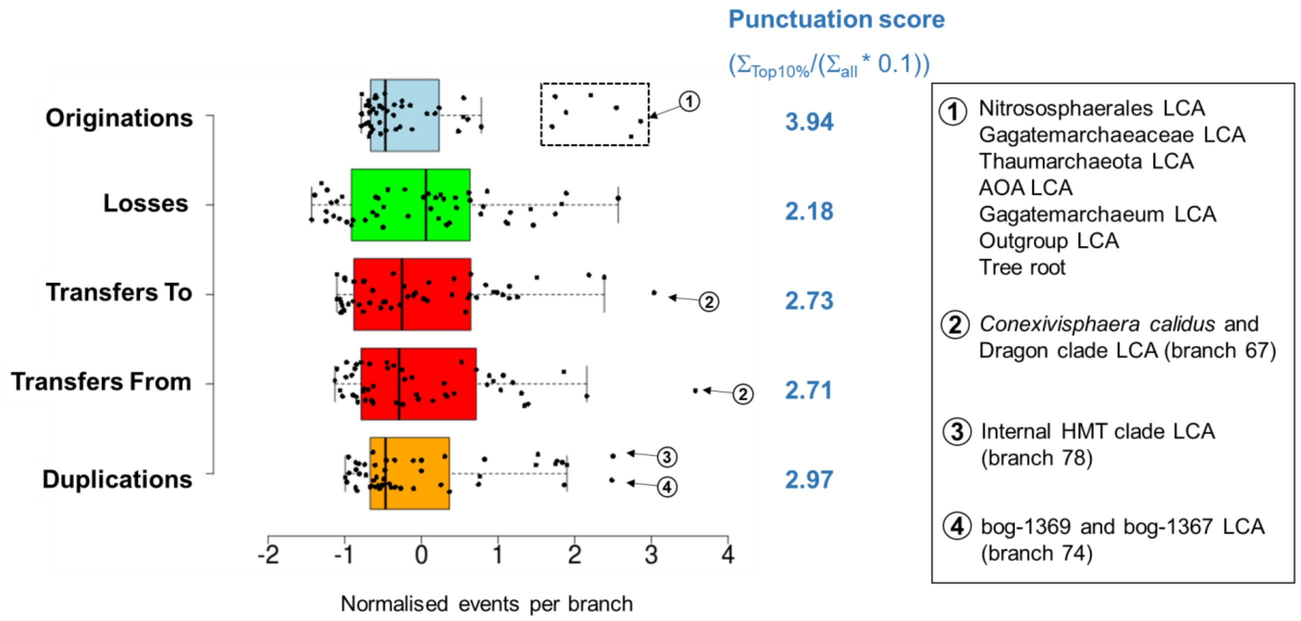

Supplementary Fig. 10. Distribution of mechanism of gene content change across Thaumarchaeota evolution. Boxplots represent normalised events per branch ((events per branch - μ)/σ) for each mechanism. Numbered circles mark ancestors (species tree branches) with the highest numbers of events. Horizontal lines within boxes indicate the medians, box boundaries indicate the 1st and 3rd quartiles, whiskers indicate the minima and maxima, and points beyond these whiskers are outliers. A punctuation score is measured for each given mechanism. It represents the sum of events in the 10% of branches with the highest event numbers divided by 10% of the sum of events into all branches ($\Sigma_{\text{events in top10\%}}/(\Sigma_{\text{events in all branches}} * 0.1)$). Source data are provided as a Source Data file.

Supplementary References

1. Vico Oton, E., Quince, C., Nicol, G. W., Prosser, J. I. & Gubry-Rangin, C. Phylogenetic congruence and ecological coherence in terrestrial Thaumarchaeota. *The ISME journal* **10**, 85-96 (2016).
