# Supplementary material for: Genomics of soil depth niche partitioning in the Thaumarchaeota family Gagatemarchaeaceae
Source: Nat Commun. 2023 Nov 11;14:7305. doi: 10.1038/s41467-023-43196-0 (PMC10640624; doi:10.1038/s41467-023-43196-0)
Supplement: Supplementary file 3 — Description of Additional Supplementary Files [file 41467_2023_43196_MOESM3_ESM.pdf]

**Supplementary Data 1**	Genome descriptions and characteristics
**Supplementary Data 2**	Pairwise comparison of average amino acid identity of Thaumarchaeota and closely related genomes
**Supplementary Data 3**	Pairwise comparison of 16S rRNA gene similarity of Thaumarchaeota and closely related genomes
**Supplementary Data 4**	Presence of Group I.1c in >150,000 16S rRNA libraries
**Supplementary Data 5**	Presence of topsoil and subsoil Group I.1c lineages in >150,000 16S rRNA libraries
**Supplementary Data 6**	Sites sampled for metagenomic sequencing
**Supplementary Data 7**	Competitive read recruitment of metagenomic reads against Group I.1c genomes
**Supplementary Data 8**	Vico Oton Group I.1c 16S rRNA gene classification of Gagatemarchaeaceae genomes
**Supplementary Data 9**	Group I.1c genomes as type material for reclassification
**Supplementary Data 10**	Key pathways in Thaumarchaeota genomes
**Supplementary Data 11**	Synteny analysis of genes surrounding rbcL, using AcS1-13 gene order as the reference
**Supplementary Data 12**	Total carbohydrate active enzymes (CAZymes) of the Thaumarchaeota
**Supplementary Data 13**	Secreted carbohydrate active enzymes (CAZymes) of the Thaumarchaeota
**Supplementary Data 14**	Total peptidases of the Thaumarchaeota
**Supplementary Data 15**	Secreted peptidases of the Thaumarchaeota
**Supplementary Data 16**	Reconciliation statistics of F420-dependent glucose-6-phosphate dehydrogenase gene family
**Supplementary Data 17**	GenBank accessions of expanded inter-domain set of prokaryotic genomes
**Supplementary Data 18**	Locus tags of pyrroloquinoline quinone-dependent dehydrogenases
**Supplementary Data 19**	Quantified mechanisms of gene content change
**Supplementary Data 20**	Duplication and loss of gene families originating in Gagatemarchaeaceae LCA
**Supplementary Data 21**	Major donors of laterally acquired genes into Gagatemarchaeaceae LCA
**Supplementary Data 22**	Genomes present in each of the three genome datasets
