# Supplementary material for: Genomics of soil depth niche partitioning in the Thaumarchaeota family Gagatemarchaeaceae
Source: Nat Commun. 2023 Nov 11;14:7305. doi: 10.1038/s41467-023-43196-0 (PMC10640624; doi:10.1038/s41467-023-43196-0)
Supplement: Supplementary file 5 — Reporting Summary [file 41467_2023_43196_MOESM5_ESM.pdf]

nature portfolio

Corresponding author(s): Cécile Gubry-Rangin

Last updated by author(s): Oct 9, 2023

Reporting Summary

Nature Portfolio wishes to improve the reproducibility of the work that we publish. This form provides structure for consistency and transparency in reporting. For further information on Nature Portfolio policies, see our Editorial Policies and the Editorial Policy Checklist.

Statistics

For all statistical analyses, confirm that the following items are present in the figure legend, table legend, main text, or Methods section.

n/a	Confirmed	
☒	☐	The exact sample size (*n*) for each experimental group/condition, given as a discrete number and unit of measurement
☒	☐	A statement on whether measurements were taken from distinct samples or whether the same sample was measured repeatedly
☐	☒	The statistical test(s) used AND whether they are one- or two-sided<br>*Only common tests should be described solely by name; describe more complex techniques in the Methods section.*
☒	☐	A description of all covariates tested
☒	☐	A description of any assumptions or corrections, such as tests of normality and adjustment for multiple comparisons
☒	☐	A full description of the statistical parameters including central tendency (e.g. means) or other basic estimates (e.g. regression coefficient) AND variation (e.g. standard deviation) or associated estimates of uncertainty (e.g. confidence intervals)
☐	☒	For null hypothesis testing, the test statistic (e.g. *F*, *t*, *r*) with confidence intervals, effect sizes, degrees of freedom and *P* value noted<br>*Give P values as exact values whenever suitable.*
☒	☐	For Bayesian analysis, information on the choice of priors and Markov chain Monte Carlo settings
☒	☐	For hierarchical and complex designs, identification of the appropriate level for tests and full reporting of outcomes
☒	☐	Estimates of effect sizes (e.g. Cohen's *d*, Pearson's *r*), indicating how they were calculated

Our web collection on statistics for biologists contains articles on many of the points above.

Software and code

Policy information about availability of computer code

Data collection	No software was used for data collection.
Data analysis	Custom scripts have been deposited at https://github.com/Tancata/phylo/tree/master/ALE (https://doi.org/10.5281/zenodo.4012549), https://github.com/SheridanPO-Lab/I.1c-Group (https://doi.org/10.5281/zenodo.8421019) and https://github.com/SheridanPO/ALE_analysis (https://doi.org/10.5281/zenodo.8421034). Open source software used in analysis is referenced in Materials and Methods: MEGAHIT v1.1.3, bwa-mem v0.7.17, MaxBin2, metaBAT2, Prokka v1.14, CheckM v1.1.2, QUAST v5.0.2, Tome v2.0, Barrnap v0.9, GTDB-Tk v1.3.0, Roary v3.12.0, MAFFT v7.407, Trimal v1.4.1, PHItest v1.1, IQ-TREE v2.0.3, ALE v1.0, iTOL, CompareM v0.1.1, GhostKOALA, HMMER v3.2.1, CoverM v0.6.1, Diamond v0.9.28, BLASTn v2.9.0.

For manuscripts utilizing custom algorithms or software that are central to the research but not yet described in published literature, software must be made available to editors and reviewers. We strongly encourage code deposition in a community repository (e.g. GitHub). See the Nature Portfolio guidelines for submitting code & software for further information.

Data

Policy information about availability of data

All manuscripts must include a data availability statement. This statement should provide the following information, where applicable:

- Accession codes, unique identifiers, or web links for publicly available datasets
- A description of any restrictions on data availability
- For clinical datasets or third party data, please ensure that the statement adheres to our policy

Accession numbers for the 15 new genomes presented in this study can be found in Supplementary Data 1 and under the NCBI BioProject PRJNA883052 (https://www.ncbi.nlm.nih.gov/bioproject/PRJNA883052/). The accession numbers for publicly available genome sequences used in the phylogenomic genome datasets can be found in Supplementary Data 22 and accessions for the expanded inter-domain set of prokaryotic genomes, used for single gene tree analysis, can be found in Supplementary Data 17. Public data is available from NCBI (www.ncbi.nlm.nih.gov), KEGG (https://www.genome.jp/kegg/), dbCAN (http://bcb.unl.edu/dbCAN2/download/), arCOG (https://ftp.ncbi.nih.gov/pub/wolf/COGs/arCOG/), PFAM(https://pfam.xfam.org/), TIGRFAM (http://tigrfams.jcvi.org/cgi-bin/index.cgi) and GTDB R202 (https://data.gtdb.ecogenomic.org/releases/).

Research involving human participants, their data, or biological material

Policy information about studies with human participants or human data. See also policy information about sex, gender (identity/presentation), and sexual orientation and race, ethnicity and racism.

Reporting on sex and gender	n/a
Reporting on race, ethnicity, or other socially relevant groupings	n/a
Population characteristics	n/a
Recruitment	n/a
Ethics oversight	n/a

Note that full information on the approval of the study protocol must also be provided in the manuscript.

Field-specific reporting

Please select the one below that is the best fit for your research. If you are not sure, read the appropriate sections before making your selection.

☐ Life sciences ☐ Behavioural & social sciences ☒ Ecological, evolutionary & environmental sciences

For a reference copy of the document with all sections, see nature.com/documents/nr-reporting-summary-flat.pdf

Ecological, evolutionary & environmental sciences study design

All studies must disclose on these points even when the disclosure is negative.

Study description	Presentation and genomic analysis of novel metagenome-assembled genomes sequences from the deeply-rooted Thaumarchaeota and evolutionary analysis of the phylum by gene tree - species tree reconciliation techniques, with an emphasis on the understudied early diverging lineages.
Research sample	Analysis involved taxonomically diverse public genome sequences and 15 novel deeply-rooted Thaumarchaeota genomes from this study. The novel genome sequences were sequenced from acidic soils collected around Scotland, UK. The publicly available genome sequences were sequenced from a wide variety of environmental sources, including marine, soil and hot spring environments. The rationale of this sampling was intended to represent the Thaumarchaeota phylum with related outgroups, so that genome evolution in the major lineages of Thaumarchaeota could be investigated, with a particular emphasis on the novel family Gagatemarchaeaceae. Publicly available genomes were genomes were downloaded from NCBI (www.ncbi.nlm.nih.gov).
Sampling strategy	Gene duplication, transfer, loss and origination data was sampled from 51 branches. This sample size is the total number of internal branches on the Thaumarchaeota species tree used for gene tree-species tree reconciliation. This sample size was set by number of branches on the species tree. All information was sampled from all internal branches, rather than a statistically calculated subset.
Data collection	Public Thaumarchaeota genome sequences were downloaded from NCBI (www.ncbi.nlm.nih.gov) by Dr Paul O. Sheridan onto a local computer system.
Timing and spatial scale	Collection of data from public repositories was conducted (started and stopped, one collection) on 11 January 2022. No further public genomes were utilized after this date, as due to the nature of the data analysis in this study (the formation of gene families, construction of gene trees and reconciliation against species trees) it was not possible to add additional genomes without restarting the entire analysis. This is the rationale for a single data collection and no subsequent sampling.

Data exclusions	Genomes with a completeness lower than 45 % or contamination greater than 10 % were excluded from the study. A stricter threshold was applied to genomes used in gene tree - species tree reconciliations. In this case, genomes with a completeness lower than 70 % or contamination greater than 5 % were excluded<br>These thresholds were chosen specifically for this dataset.
Reproducibility	Code and databases used to analyze data in the study are now publicly available as described in the code and data availability sections, enabling the results to be reproduced by anyone proficient in genomics and with access to adequate computational resources
Randomization	The genomes were analyzed as a single group without partitions. Genes encoded by genomes were related to each other by amino acid sequence information, rather than by any a priori clustering and can thus be considered to be randomized
Blinding	The genomes were analyzed as a single group with no a priori clustering. While researchers were not blind to the taxonomy of the genomes, this information was not provided to the phylogenetics software used to infer the phylogenies of genes and species.

Did the study involve field work? ☐ Yes ☒ No

Reporting for specific materials, systems and methods

We require information from authors about some types of materials, experimental systems and methods used in many studies. Here, indicate whether each material, system or method listed is relevant to your study. If you are not sure if a list item applies to your research, read the appropriate section before selecting a response.

Materials & experimental systems

n/a	Involved in the study
☒	☐ Antibodies
☒	☐ Eukaryotic cell lines
☒	☐ Palaeontology and archaeology
☒	☐ Animals and other organisms
☒	☐ Clinical data
☒	☐ Dual use research of concern
☒	☐ Plants

Methods

n/a	Involved in the study
☒	☐ ChIP-seq
☒	☐ Flow cytometry
☒	☐ MRI-based neuroimaging

Plants

Seed stocks	n/a
Novel plant genotypes	n/a
Authentication	n/a
